# Room-Temperature Single-Photon Sources Based on Colloidal Quantum Dots: A Review

**DOI:** 10.3390/ma16247684

**Published:** 2023-12-17

**Authors:** Yongzheng Ye, Xing Lin, Wei Fang

**Affiliations:** 1 Interdisciplinary Center for Quantum Information, State Key Laboratory of Extreme Photonics and Instrumentation, College of Optical Science and Engineering, Zhejiang University, Hangzhou 310027, China; yyzidea@zju.edu.cn; 2 College of Information Science and Electronic Engineering, Zhejiang University, Hangzhou 310027, China; lxing@zju.edu.cn; 3 Key Laboratory of Excited-State Materials of Zhejiang Province, Zhejiang University, Hangzhou 310027, China; 4 Jiaxing Key Laboratory of Photonic Sensing & Intelligent Imaging, Jiaxing 314000, China; 5 Intelligent Optics & Photonics Research Center, Jiaxing Research Institute Zhejiang University, Jiaxing 314000, China

**Keywords:** single-photon sources, colloidal quantum dots, quantum photonics

## Abstract

Single-photon sources (SPSs) play a crucial role in quantum photonics, and colloidal quantum dots (CQDs) have emerged as promising and cost-effective candidates for such applications due to their high-purity single-photon emission at room temperature. This review focuses on various aspects of CQDs as SPSs. Firstly, a brief overview of the fundamental optical properties of CQDs is provided, including emission wavelength engineering and fluorescence intermittency, and their single-photon emission properties. Subsequently, this review delves into research concerning CQDs as SPSs, covering topics such as the coupling of single CQDs to microcavities, both in weak and strong coupling regimes. Additionally, methods for localizing and positioning CQDs are explored, which are critical for on-chip SPSs devices.

## 1. Introduction

The development of photonic quantum information technologies, such as quantum key distribution [1,2,3], photonic quantum computing [4], and quantum metrology [5,6], demands single-photon sources (SPSs) or entangled photon-pair sources. An ideal SPS emits exactly one photon at a time into a given polarization and spatiotemporal mode [7]. It cannot be prepared from a classical light source, as either a coherent or a thermal light source may generate multi-photons with certain probability, no matter how such a source is attenuated. To date, the most widely used SPSs are based on spontaneous parametric down-conversion [8,9,10] or spontaneous four-wave mixing [11,12,13]. However, the nonlinear processes occur randomly. Moreover, the probability of generating multi-pair events increases with pump power, which restricts the brightness of the SPSs. A more promising type of SPS is based on spontaneous emission from a two-level system. Once it relaxes from the excited state and emits one photon, such quantum system is unable to re-emit immediately, thus inherently being single-photon-like. Moreover, the single-photon purity is intrinsically decoupled from pumping strength. SPSs based on single quantum emitters have been demonstrated in systems such as atoms [14], ions [15], molecules [16], defect states [17,18], semiconductor epitaxial quantum dots (EQDs) [19], and colloidal quantum dots (CQDs) [20], among which solid state emitters have the advantages of scalability and integration.

With rapid progresses in the past few decades, EQDs embedded in microcavities can generate near-ideal single-photons in terms of single-photon purity, brightness, indistinguishability, and polarization [21]. However, the best performance sources based on InGaAs quantum dots (QDs) only work at cryogenic temperature, limiting practical applications. Moreover, the undesirable features of the self-organizing technique such as random sizing and positioning have hindered the EQDs from scalable applications. On the other hand, CQDs, or semiconductor nanocrystals, synthesized using solution-based methods, offer a viable solution for room-temperature applications. Excitingly, the Nobel Prize in Chemistry 2023 was awarded to Moungi G. Bawendi, Louis E. Brus, and Aleksey I. Yekimov for their discovery and synthesis of CQDs. This recognition is expected to further stimulate exploration into the applications of CQDs. In fact, CQDs have already been demonstrated as a low-cost solution characterized by bright and stable room-temperature single-photon emission [20,22,23]. Size- and composition-dependent emission of CQDs can cover a wide spectral range from ultra-violet to near-infrared [24]. During the past ∼30 years, synthetic chemistry of CQDs has advanced substantially, allowing superior control of their emission properties. The sizes of CQDs can be precisely controlled by the synthetic processes. Near-unity photoluminescence (PL) quantum yield (QY) is confirmed for a number of material systems of CQDs [25]. The troublesome PL blinking of a single CQD, switching between different brightness states under constant optical excitation, is greatly suppressed down to ∼10^−6^ per photon absorption for the well-developed CdSe/CdS core/shell CQDs [26,27]. Moreover, the compatibility of the solution process makes the device fabrication cheap and flexible, and the location of individual CQDs can be manipulated easily with high accuracy as well (see Section 5). These facts are great encouragements to utilize high-quality CQDs as room-temperature quantum emitters in SPSs. However, so far, only a few review articles have specifically addressed this topic [28,29,30].

In this review, the related topics about CQDs as SPSs are discussed. The background on the optical properties of CQDs is provided, including emission wavelength engineering, fluorescence intermittency, and their single-photon emission properties. Research on coupling individual CQDs to photonic structures to modify the spontaneous emission through Purcell enhancement are summarized. Microcavities can optimize CQD SPS performance, increasing collection efficiency, accelerating emission rates, and narrowing linewidths. Plasmonic nanocavities further enable strong coupling between CQDs and plasmons for studying light–matter interactions. Finally, progresses in positioning and immobilizing single CQDs on photonic chips are highlighted, a requirement for integrated SPS applications.

## 2. Optical Properties of Colloidal Quantum Dots

### 2.1. Emission Spectrum Engineering in Colloidal Quantum Dots

**Figure 1:**
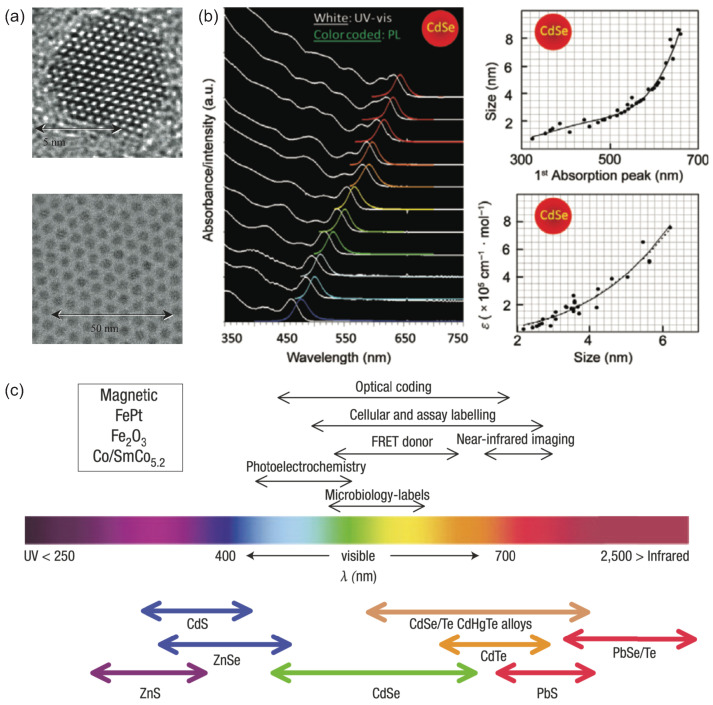
(**a**) Transmission electron microscopy (TEM) image of typical CdSe CQDs [38]. Reproduced with permission from Reference [38]. Copyright 2002 the Royal Society. (**b**) The size-dependent absorption, PL spectrum, and extinction coefficient of CdSe CQDs. The sizes of CdSe CQDs in absorption and PL spectrum are 1.6, 2.1, 2.3, 2.4, 2.9, 3.3, 3.6, 4.3, 4.6, 5.1, and 6.2 nm, respectively, from bottom to top [39]. Reproduced with permission from Reference [39]. Copyright 2009 Springer Nature. (**c**) II–VI and IV–VI based CQD materials scaled as their emission wavelength coverage [24]. Reproduced with permission from Reference [24]. Copyright 2005 Springer Nature.

CQDs, typically synthesized and processed in the solution phase, are nanometer-sized semiconductor crystals with typical dimensions below 10 nm, as shown in Figure 1a. The most intriguing property of CQDs is the size-dependent energy bandgap due to the quantum confinement effect, which was proposed by L. Brus and A. Efros in their pioneering works in the early 1980s [31,32]. As shown in Figure 1b, the absorption and emission spectra depend strongly on the CQD sizes. After the breakthrough work by Henglein in 1982 [33], the field of synthesis chemistry of CQDs developed into a more controllable, quantitative and understandable stage in 1990s, represented by the CdSe system developed by Brus’s group [34] and Bawendi’s group [22]. In this system, the concept of ‘‘focusing of size distribution’’ was formulated, which makes directly synthesizing CQDs with nearly monodisperse possible [35].

A broader emission wavelength tuning range can be achieved by modifying the elementary composition or engineering the energy potential profile [24,36]. As shown in Figure 1c, CQDs made of II–VI and IV–VI semiconductor compounds, such as ZnS, CdS, ZnSe, CdSe, CdTe, and PbSe, exhibit emission spectra covering a wide range from ultraviolet to near-infrared wavelengths. Among these compounds, CdSe CQDs, which span a significant portion of the visible spectrum, have undergone the most substantial development. On the other hand, PbSe CQDs demonstrate emission overlapping with the 1.5 μm telecommunications window [37].

Furthermore, an alternative method for bandgap engineering involves epitaxially growing a semiconductor shell layer with a wide band gap [24,40]. This allows control over the size, shape, and composition of the CQDs. The presence of this shell layer is crucial for maintaining a high QY, as discussed in the next section. Detailed discussion on the material composition, structural design, and size of CQDs, which are intimately linked to their specific internal energy level structures, can be found in References [40,41].

### 2.2. Photoluminescence Intermittency of Single Colloidal Quantum Dots

**Figure 2:**
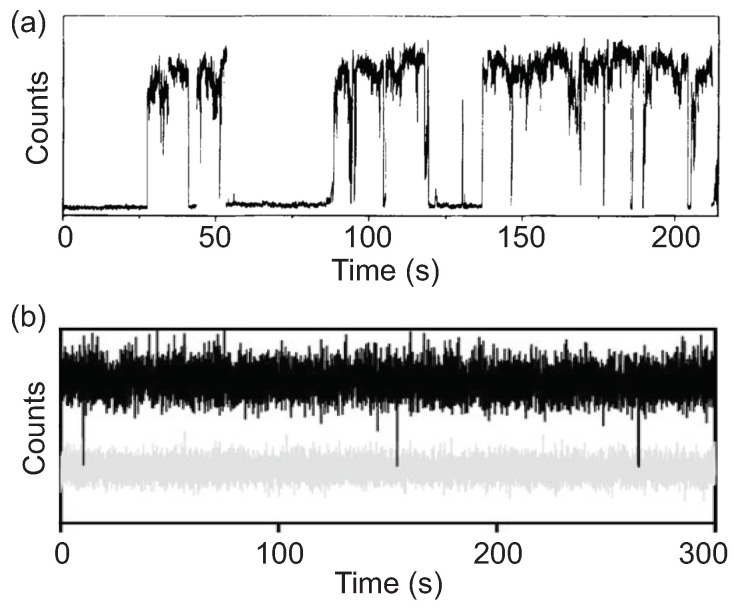
PL intensity time traces of (**a**) a blinking single CQD [47] and (**b**) a non-blinking CQD [26]. Here the gray trace is the background noise intensity.Panel a is reproduced with permission from Reference [47]. Copyright 1996 Springer Nature. Panel b is reproduced with permission from Reference [26]. Copyright 2014 American Chemical Society.

CQDs possess broad absorption bands, narrow and tunable emission, and excellent photostability at room temperature, making them powerful light-emitting materials in the nanoscale region, especially for biotechnologies. However, a major obstacle for single-dot applications is the random fluctuation of emission intensity, known as PL blinking (Figure 2a). When a single CQD is optically excited, its emission randomly switches between a bright “on” state and a dark “off” state [42,43]. PL blinking has been extensively studied for more than 20 years and is widely attributed to surface traps and Auger processes [44,45,46]. After a hot electron and hole pair is generated in a CQD, it quickly relaxes to form a band-edge exciton. If this exciton recombines radiatively, it emits a photon. However, if a defect state is present in the CQD, it can trap one of the carriers, leading to the formation of a charged exciton when another exciton is excited. Due to the significant Coulomb interaction between carriers in the low-index dielectric environment and the small size of the CQD, the charged exciton undergoes an efficient non-radiative relaxation pathway known as the Auger process. In this charged exciton state, the recombination energy is consumed without photon emission, leading to lower emission intensity, lower QY, and shorter PL lifetime until the free carrier is neutralized.

In contrast to EQDs, which are typically embedded in a defect-free crystalline environment, CQDs possess abundant active sites associated with surface atoms that have unpaired electrons or unfilled orbitals. Once an exciton is formed in a CQD, it is susceptible to the crystal boundary where the cationic/anionic sites work as electron/hole traps [48]. Consequently, careful surface passivation is essential for CQDs to maintain high QY. In general, the emissive core is epitaxially coated with wide band gap semiconductors to form core/shell heterostructure [49,50,51,52,53,54,55,56,57]. The inorganic shell saturates the surface dangling bonds and isolates both electron and hole wavefunctions from the outer surface by creating an energy potential barrier. Although shell growth significantly improves PL QY, only CQDs with extremely thick shells initially showed suppressed blinking [58,59]. Apart from inorganic shell, there are a variety of organic ligands for the surface passivation of CQDs [60], such as fatty acids [61], amines [61,62], thiols [63], and so on. The presence of outer ligands not only enables solution processibility of nanocrystals but also passivates dangling bonds and surface traps, leading to near-unity quantum yield. Additionally, the ligands used, such as fatty amines and fatty acids, can be functionalized or exchanged to suit different solvent systems [48]. Recent developments of phase pure CQDs have broken the nonblinking volume threshold, as shown in Figure 2b, and the emission spectra of CQDs cover most part of the visible window [26,36,64]. The switching rate between bright and dark state can be as low as ∼$10^{-6}$ per photon absorption, coupled with a spontaneous discharging process with a rate of ∼2 events/s [27].

## 3. Single-Photon Generation from Colloidal Quantum Dots

### 3.1. Photon Statistics Measurement of a Light Source

**Figure 3:**
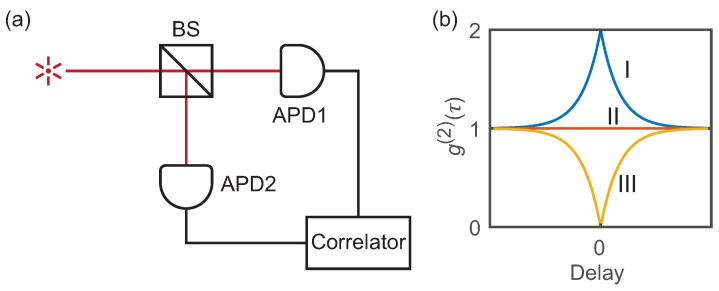
(**a**) The Hanbury Brown and Twiss setup for photon statistics study. (**b**) The second-order coherence function $g^{\left(2\right)}\left(\tau \right)$ versus interphoton delay τ for (I) bunched (thermal), (II) coherent (laser), and (III) antibunched light sources.

Photon statistical measurement is required to verify the quantum property of a light source. An ordinary single-photon detector (SPD) that operates at Geiger mode is unable to resolve the number of photons arriving at a time, as it cannot respond to another incoming photon during the several tens of nanoseconds long dead time period after the first detection. Therefore, studying the photon statistics of a light source cannot be accomplished using a single SPD. A Hanbury Brown and Twiss (HBT) setup is typically employed, where two independent detectors are deployed at two output ports of a beam splitter [65], as shown in Figure 3a. In this arrangement, the detector on the second arm can register the incoming photon event while the first one is still in a dead state. The time difference (τ) between the detection events of the two channels (referred to as start and stop) is calculated and recorded to generate the histogram of coincidence events as a function of τ as derived by Reynaud [66]. The second-order correlation function $g^{\left(2\right)}\left(\tau \right)$ can then be obtained from the histogram by normalization.

The second-order correlation function $g^{\left(2\right)}\left(\tau \right)$ that represents the degree of correlation between the number of photons detected at time *t* and at time t+τ is defined as:

(1) $$g^{\left(2\right)}\left(\tau \right)=\frac{\langle n(t)n(t+\tau )\rangle }{{\langle n(t)\rangle }^{2}},$$

where n(t) is the number of photons counted at time *t*. When τ=0, $g^{\left(2\right)}$ reflects the statistics property of the light source. As shown in Figure 3b, the number of photons follows Poisson distribution for a coherent light (laser) source, which results in $g^{\left(2\right)}\left(0\right)=1$. And a conventional incoherent source, or thermal light, follows the super-Poisson distribution and has $g^{\left(2\right)}\left(0\right)=2$. For an ideal single-photon source, $g^{\left(2\right)}\left(0\right)$ goes to 0. In short, the possibility of two or more photons to be detected simultaneously by the two detectors is zero.

### 3.2. Colloidal Quantum Dots as Single-Photon Emitters

Single-photon emission from a single CQD was firstly demonstrated independently by P. Michler [20] and B. Lounis [67] in their pioneering works. Since then, CQDs have garnered significant interest in the single-photon source research community due to their unique properties, including high QY at room temperature, broad absorption spectra, and photochemical stability under moderate ultraviolet irradiation.

In principle, an ideal quantum emitter based on a two-level system will generate light with $g^{\left(2\right)}\left(0\right)=0$. However, in the case of a QD system, biexciton are inevitably generated even under low-excitation conditions, and the cascade two-photon emission from biexciton state can impair the single-photon purity. For EQDs, the emission linewidths are much smaller than the binding energy of biexciton at cryogenic temperature, allowing the photons generated from excitons to be isolated using a narrow-band filter [68]. However, CQDs have much broader room-temperature emission linewidths, usually >50 meV, rendering filtering ineffective due to spectral overlap. Fortunately, CQDs usually have much smaller dimensions, typically less than 10 nm. The strong confinement of carrier wavefunctions significantly enhances the Auger effect, which quenches the biexciton quantum yield by less than a few percent, thereby ensuring single-photon purity [69].

### 3.3. Polarized Single-Photon Generation from Collodial Quantum Dots

**Figure 4:**
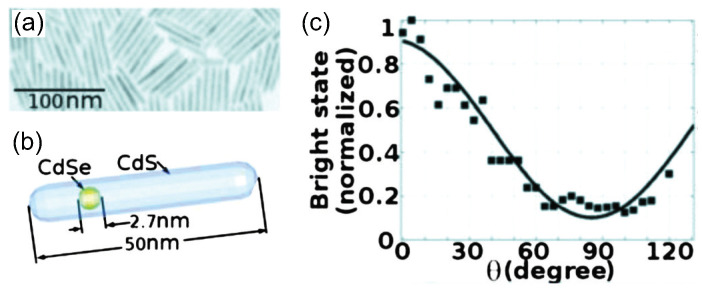
TEM image (**a**) and illustration (**b**) of the synthesized dot-in-rod CQDs. (**c**) Mean value of the bright state as a function of the polarization detection angle [74]. Reproduced with permission from Reference [74]. Copyright 2010 AIP Publishing.

Polarization properties are crucial factors for SPSs, especially in quantum cryptography protocols like BB84 and B92 [1,2,3], where cryptographic keys are encoded on the polarization of single photons. For such applications, SPSs with deterministic photon polarization are required. This requirement can be met by modifying the shape of CQDs, such as elongated CdSe CQDs [70] or embedding a CdSe core in an elongated CdS shell [71,72,73], as shown in Figure 4b.

The linearly polarized emission from CQDs was firstly demonstrated in elongated CdSe quantum rods by Hu et al. [70]. By increasing the aspect ratio (length to diameter), the degree of polarization of the PL emission along the elongated direction can be increased from near zero (for spherical CQDs) to ∼70% for CQDs with an aspect ratio greater than 2. The polarization can be further enhanced by embedding an elongated core in a rod [72]. With the aspect ratio higher than 3, the degree of polarization is about 82% for CdSe/CdS rod-in-rod CQDs. The single-photon emission of dot-in-rod CQDs was observed by Pisanello et al. [74]. Figure 4 shows that single dot-in-rod CQDs with a rod length of ∼50 nm and a core diameter ∼2.7 nm exhibit a photon antibunching dip of $g^{\left(2\right)}\left(0\right)\approx 0.02$ and a degree of polarization of about 80%.

Other than CQD shape control, deterministic polarized single-photon emission can be obtained with the help of photonic structures. As a cholesteric (chiral nematic) liquid crystal (CLC) layer will reflect the light with the same rotation of the electric field vector as the rotation of CLC molecules [75], CdSeTe CQDs doped in left-handed planar-aligned CLC film can be observed to emit a significantly higher intensity of left-handed circular polarized light comparing to that of right-handed polarized light, and single-photon emission with $g^{\left(2\right)}\left(0\right)=0.382\pm 0.037$ can be observed [76].

## 4. Single-Photon Devices Based on Colloidal Quantum Dots

### 4.1. Cavity Quantum Electrodynamics Effects

CQDs exhibit excellent properties as quantum emitters for SPSs at room temperature, such as high QY, broadband absorption, excellent photochemical stability, flexible structure engineering for desired emission wavelengths, and easy device fabrication based on solution processes. However, CQDs also bear several disadvantages such as omni-directional emission, broad emission spectra, and long emission lifetime at room temperature. Fortunately, with the help of photonic micro/nano structures, especially applying cavity quantum electrodynamics (cQED) effects, these problems may be mitigated. By adjusting the local photonic environment, the dipole radiation modes can be directed into a desired single optical mode, and the emission spectrum and lifetime can also be modified consequently.

Of the many cQED effects, the Purcell effect is the most related one to SPSs. The Purcell factor $F_{P}=\gamma_{loc}/\gamma_{vac}$ represents the ratio of a quantum emitter’s spontaneous emission rate in local photonic environment to the rate in vacuum [77]. Accordingly, the fraction of the spontaneous emission that goes into one particular mode may also be modified so that the single-photon collection efficiency is enhanced. If the emitter is coupled to a microcavity with perfect spatial and spectral alignment, the Purcell factor can be expressed as:

(2) $$F_{P}=\frac{3}{4\pi^{2}}{\left(\frac{\lambda }{n_{eff}}\right)}^{3}\left(\frac{Q}{V}\right),$$

where $n_{eff}$ is effective refractive index, *Q* is the quality factor of the cavity, and *V* is the optical mode volume.

The Purcell effect mainly describes the interaction between the quantum emitter and photonic structure in the weak coupling region. When the interaction (or Q/V in number) is so large that the emitter–cavity coupling rate is greater than both cavity losses and the decoherence of the emitter, strong coupling region is reached [78]. Rabi oscillation in the optical field intensity in the time domain or Rabi splitting in the frequency domain will show up as a sign [78,79]. Nevertheless, precise positioning of single CQDs is necessary to achieve optimal coupling between CQDs and cavities, which will be discussed in Section 5.

### 4.2. Fabry–Pérot Microcavity

**Figure 5:**
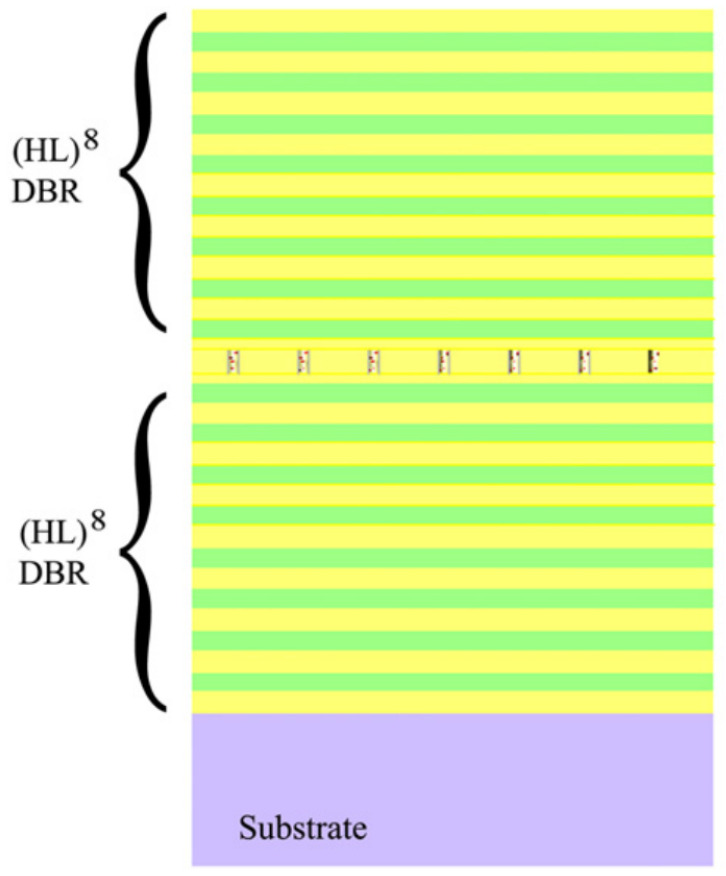
Schematic diagram of the planar cavity consisting of two ${\mathrm{SiO}}_{2}$/${\mathrm{TiO}}_{2}$ Bragg mirrors and a layer of pillars with CdSe/ZnS CQDs inside [80]. Reproduced with permission from Reference [80]. Copyright 2010 Elsevier.

The Fabry–Pérot microcavity is a typical dielectric microcavity that can be easily integrated with CQDs. A notable coupling of single CQDs to micropillars was demonstrated by Qualtieri et al. [80]. In their study, CdSe/ZnS CQDs with a central wavelength at λ=615 nm was dispersed in a negative high-resolution electron beam resist with a very low concentration. This mixture was then spin-coated onto a distributed Bragg reflector composed of eight paired ${\mathrm{SiO}}_{2}/{\mathrm{TiO}}_{2}$. Subsequently, this coated layer was sculpted into pillars, with diameters varying between 500 nm and 30 nm, through electron beam lithography. To complete the structure, another distributed Bragg reflector was constructed atop the CQD–pillar layer (as depicted in Figure 5). The smallest pillars encapsulating only single CQD exhibited single-photon emission with $g^{\left(2\right)}\left(0\right)<0.1$. The presence of cavity confinement resulted in the emission linewidth being significantly narrowed—from 30 nm down to 0.73 nm. However, the random distribution of CQDs makes it challenging for large-scale arrays where each pillar needs to contain exactly one CQD.

### 4.3. Whispering-Gallery Mode Microcavity

**Figure 6:**
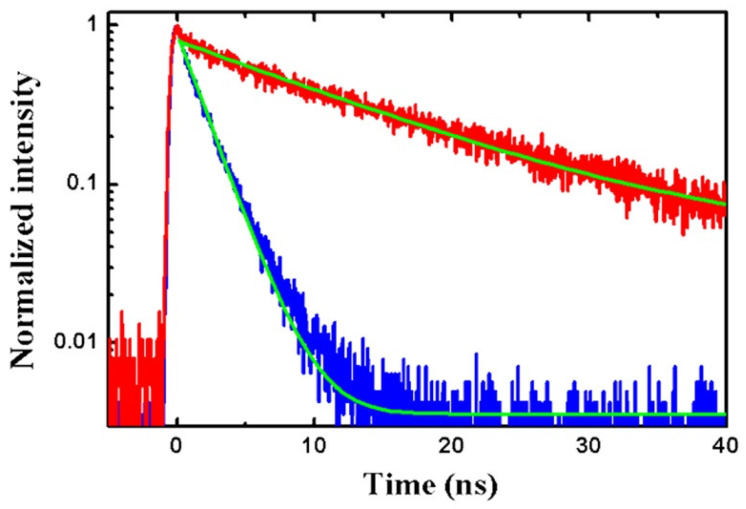
Typical fluorescence decay curves for CQDs on the edge of the disk (blue curve) and CQDs away from the disk (red curve) [82]. Reproduced with permission from Reference [82]. Copyright 2011 AIP Publishing Group.

Whispering-gallery mode microcavities with circular boundaries represent another type of resonator compatible with CQDs. Single CQDs coupled to a glass microsphere have been reported by Artemyev et al. in 2001 [81], with a maximum Purcell factor $F_{p}∼5$ at T = 20 K. At room temperature, spontaneous emission enhancement has been observed from a single colloidal CdSe/ZnS CQD located at the edge of a submicron-sized dielectric disk [82]. As shown in Figure 6, the lifetime is about 6-fold shorter than that of CQDs far away from the edge of the disk. Meanwhile, CQD laying at the disk edge exhibited an antibunching dip in the correlation function at zero time delay.

Nevertheless, both Fabry–Pérot microcavities and microdisk resonators face the same challenge of random positioning of CQDs relative to the cavity. Additionally, due to spectral diffusion, the CQD emission spectrum has a width on the order of 10 nm. Hence, a cavity with a high quality factor has small overlap between the cavity mode and CQD spectrum, leading to decreased exciton transition probability and emission rate. This diminishes the enhancement of the Purcell effect in the cavity. To date, the achieved Purcell factors are on the order of 10 for these dielectric microcavities [82].

### 4.4. Plasmonic Nanoresonator

Plasmonic nanoresonators (PNRs) are metal-based optical cavities that utilize surface plasmon polaritons [83], which offer excellent system to demonstrate prominent cQED effects with CQDs. The ultrasmall mode volume ($V\ll {\left(\lambda /n_{eff}\right)}^{3}$) enables strong coupling with CQDs, while their low quality factor results in a broad resonance that can match the room temperature emission peak of CQDs. These nanocavities have not only enabled bright single-photon emission from CQDs with large Purcell enhancement [23,84], but have also allowed the observation of vacuum Rabi splitting based on strong coupling between the CQD and cavity mode [85].

**Figure 7:**
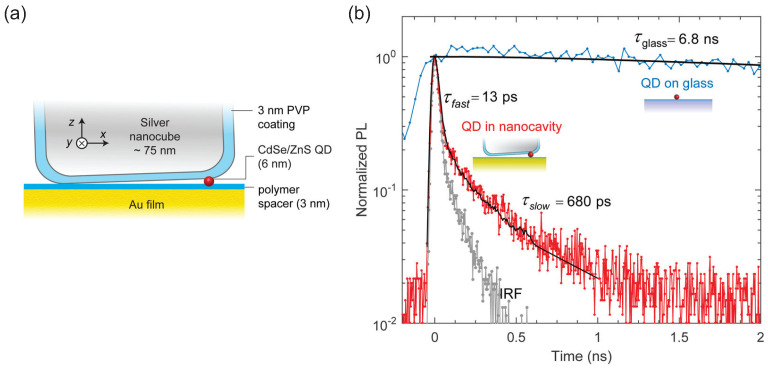
(**a**) Schematic diagram of the plasmonic nanoresonator with a CdSe/ZnS CQD in the gap region. (**b**) Time-resolved PL from a single CQD coupled to a nanocavity (red), showing a biexponential decay with a fast component of $\tau_{fast}=13$ ps and a slow component of $\tau_{fast}=680$ ps, where the fast component is limited by the instrument response function (IRF) of APD, also shown (light gray). The lifetime of a single CQD on glass is $\tau_{glass}=6.8$ ns (blue) [84]. Reproduced with permission from Reference [84]. Copyright 2015 American Chemical Society.

Figure 7a shows a PNR that consists of a 50-nm-thick gold film and a silver nanocube, with a CdSe/ZnS core/shell CQD placed in the gap [84]. Both metallic surfaces were coated with a 3 nm isolating polymer spacer layer to avoid direct contacting with the CQD. The parameters of the PNR were designed such that the cavity resonance wavelength ∼630 nm overlapped with the CQD emission spectrum.

The coupled single CQD exhibited an antibunching dip at zero delay with $g^{\left(2\right)}\left(0\right)=0.32$. The decay lifetime extracted from the curve was limited by the 250 ps time bin used in this measurement. The time-resolved PL shown in Figure 7b reflects a biexponential decay. The dominant fast component of the decay had a lifetime of 13 ps which was resonant to the nanocavity. The slow component with a lifetime of 680 ps corresponded to orthogonal emission dipoles of the CQD that did not optimally match the orientation of dominant electric field component in the cavity. The lifetime of a single CQD on glass was also measured as 6.8 ns for comparison. Considering the QYs of the single CQD coupled to a nanoresonator (50%) and CQDs on glass (20%), the Purcell factor was estimated as $F_{p}∼1350$, while numerical simulation indicated that a factor of up to 2000 could be reached with optimal coupling. A 1900-fold enhancement of PL count rate was also measured for this coupled CQD with a maximum count rate approached 1 MHz.

**Figure 8:**
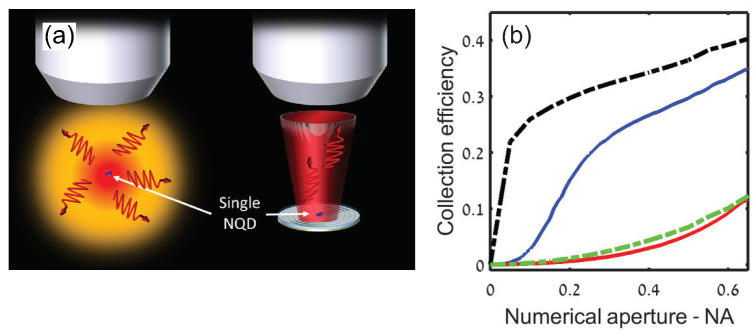
(**a**) The difference of PL emission between free-standing CQD and CQD coupled to nanoantenna. Left side: the isotropic angular emission of the CQD without a nanoantenna, resulting in a low collection efficiency. Right side: the directional emission with a nanoantenna, leading to a higher collection efficiency. NQD: nanocrystal quantum dots. (**b**) Measured photon collection efficiency as a function of the NA of the collecting lens of the CQD coupled to nanoantenna (blue) and of the reference CQD (red). The dashed black (green) lines are corresponding theoretical calculations [23]. Reproduced with permission from Reference [23]. Copyright 2016 American Chemical Society.

In addition to enhancing the spontaneous emission rate, PNR can be used to strongly modify CQD emission pattern for efficient photon collection [23]. As shown in Figure 8a, a circular bulls-eye shaped Ag grating was fabricated by E-beam lithography with a single CdTe/ZnS CQD located at the center disk. With the help of grating, photons were scattered to the normal direction, and a collection efficiency more than 35% was realized by an objective lens with a moderate NA = 0.65, which was three times better than that of the free-standing CQD with same collection system. Additionally, for a lower NA, the ratio of collection efficiency of the device to that of free-standing CQD was more significant. For instance, 20% photons could be collected by NA<0.25 optics, corresponding to the NA of a Multi-Mode fiber. This was a 21-fold enhancement compared with that of single free-standing CQD. It proved that this device was a promising scheme for high brightness SPS in compact low-NA optics. The HBT measurement was performed by pulse laser and the single-photon emission at room temperature was observed with $g^{\left(2\right)}\left(0\right)≃0.37$, which was larger than that of single free-standing CQD, which was $g^{\left(2\right)}\left(0\right)≃0.12$. The residual count at zero delay was ascribed to the biexciton emission and the weak broadband metal emission of a very short lifetime compared to the CQD PL lifetime.

When the mode volume of a PNR is further reduced, strong coupling between CQDs and PNR can be realized [85,86]. The strong coupling have various application in quantum optics and quantum information processing, such as single-photon nonlinearities for quantum gates [87], photon blockade [88,89] and stimulated Raman adiabatic passage [90].

**Figure 9:**
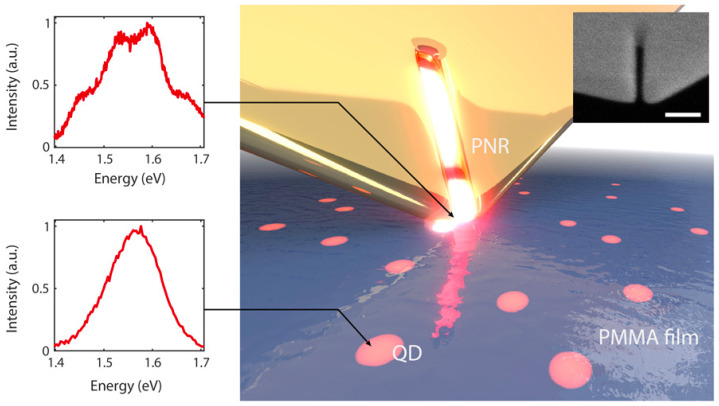
Illustration of the PNR probe interacting with single CQDs embedded in a polymer film. Left panel: the spectrum of coupled and uncoupled single CQD. Inset: scanning electron microscope (SEM) image of a nanoresonator at the apex of a probe tip. Scale bar, 100 nm [85]. Reproduced with permission from Reference [85]. Copyright 2018 American Association for the Advancement of Science.

As shown in Figure 9, a slit-like PNR probe was fabricated at a corner of a single-crystal gold flake to couple with individual non-spherical CdSeTe/ZnS CQDs in PMMA film [85]. Scanning probe technology was applied to reliably and repeatedly position single CQDs with nanometer precision beneath the slit opening. With the help of a broad resonance range, plasmon could simultaneously interact with all band-edge states of asymmetrical CQDs, enhancing the electric dipole moment from 5 Debye to 15 Debye. When the CQD was positioned beneath the tip apex, a pronounced short-range confinement emerged between the CQD and the PNR’s apex. This led to a significantly reduced plasmonic field mode volume compared to an isolated PNR without a proximal CQD. The near-field proximity effect resulted in a more compact mode volume, and consequently, a stronger coupling strength. With a relatively wide bandwidth of 78 meV, the PNR could simultaneously couple with the charged and neutral QD states. Consequently, four peaks emerged, exhibiting strong coupling strengths of 110 meV and 44 meV for the neutral and charged excitons, respectively.

### 4.5. Photonic Nanowire

Although coupling quantum dots with microcavities can achieve large Purcell effects and increase single-photon collection efficiency, the spatial and spectral alignment poses high requirements for these applications. In contrast, photonic nanowires with subwavelength diameter can alter the radiation pattern of a quantum emitter on its surface by tightly confining the optical field, thus increasing single-photon collection efficiency. Despite little change on the emission rate, the broadband and position-insensitive coupling between the photonic nanowire and CQD enables more convenient implementation for applications.

**Figure 10:**
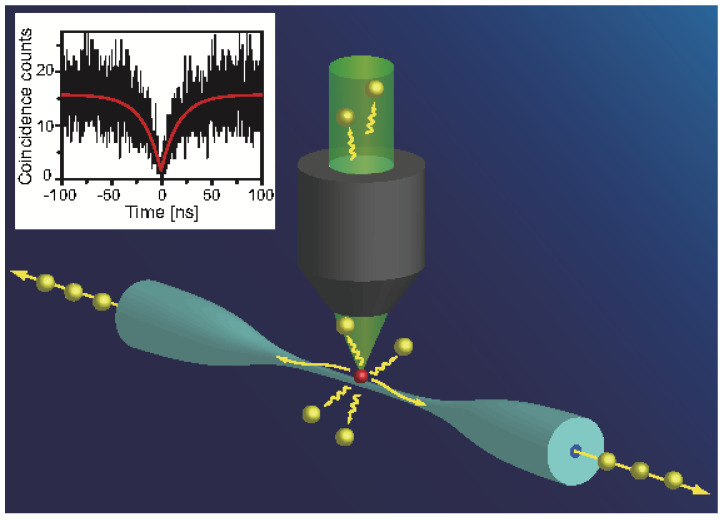
Schematic diagram of a single CQD coupled into a tapered fiber. The inset is the $g^{\left(2\right)}\left(\tau \right)$ curve of single coupled CQD [92]. Reproduced with permission from Reference [92]. Copyright 2011 American Chemical Society.

A simple way to produce photonic nanowires without sophisticated equipment is by just elongating a conventional optical fiber to reach an optical nanofiber diameter in the hundreds of nanometers range [91]. Meanwhile, the taper region naturally formed during the stretching process enables efficient coupling between the nanofiber and the single mode optical fiber. SPSs have been realized with relatively high collection efficiency by depositing CQDs on the surface of the nanofiber and optically exciting a single dot [92,93]. As shown in Figure 10, a simple nanofiber structure enabled measurement of a single-photon collection efficiency around 7.4% [92]. This value was later improved to 22.0% [94], approaching the theoretical limit around 30% [95].

**Figure 11:**
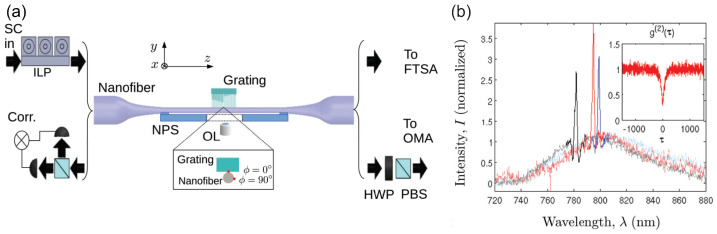
(**a**) Conceptual schematic diagram and design parameters of the device consisting of nanofiber and nanofabricated grating. (**b**) Measured PL intensity spectra for three different single CQDs. The inset shows a typical $g^{\left(2\right)}\left(\tau \right)$ curve [96]. Reproduced with permission from Reference [96]. Copyright 2014 American Physical Society.

The coupling between photonic nanowires and CQDs can be further enhanced by incorporating nearby photonic structures, although at the cost of precise positioning requirements. As shown in Figure 11, a single CQD on the surface of an optical nanofiber was optically contacted with a nano-grating [96]. This enabled the on-resonance region of the CQDs’ PL spectrum to be enhanced by up to 15-fold compared to the off-resonance region. The correlation function at zero delay $g^{\left(2\right)}\left(0\right)$ was 0.3 for on-resonance photons, indicating single-photon emission. Alternatively, Kolchin et al. positioned single CQDs inside a low index air gap between a high-index Si nanowire and ZnS slab [97]. The discontinuity in refractive indices induced strong electric field confinement in the gap along the nanowire, enhancing the spontaneous decay rate by 31-fold.

### 4.6. Electrical Driven Single-Photon Source

Most CQD-based SPSs utilize optical excitation, lending simplicity to the device structure. However, in on-chip integrated photonic quantum applications—particularly when both the quantum light source and detector reside on the same chip—the excitation light from optical methods can introduce pronounced background noise, compromising device performance. This underscores the appeal of electrically driven single-photon sources, which offer a pristine environment in integrated quantum applications, especially under room temperature conditions [98].

**Figure 12:**
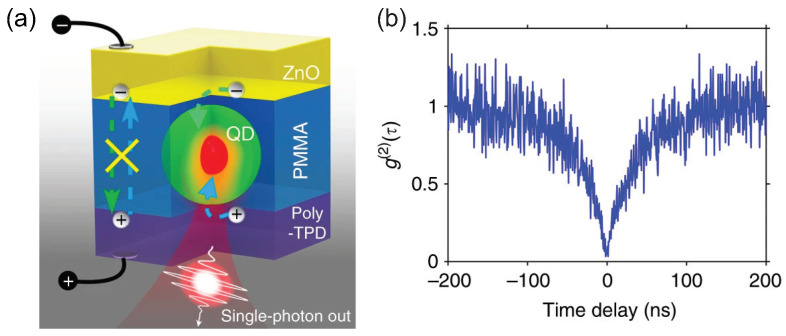
(**a**) Schematic illustration of device structure. (**b**) $g^{\left(2\right)}\left(\tau \right)$ curve of a quantum dot driven at 2.6 V [99]. Reproduced with permission from Reference [99]. Copyright 2017 Springer Nature.

The pioneering demonstration of electrically excited single-photon sources operating at room temperature using CQDs was presented by Lin et al. [99]. In their study, CdSe/CdS CQDs were embedded within a PMMA layer, its thickness slightly exceeding the diameter of the CQDs, as depicted in Figure 12a. This PMMA layer was sandwiched by between a Poly-TPD layer, serving as the hole-transport layer, and a layer of ZnO nanoparticles designated as the electron-transport layer. The insulating role of the PMMA layer was crucial to the device’s performance. It facilitated the injection or tunneling of electrons and holes into the CQDs while thwarting the direct recombination of electrons and holes in the carrier transport layers. Furthermore, the controlled dynamics of the carriers curtailed the formation of the biexciton state, enhancing the purity of the emitted single-photons. Collectively, these factors led to an impressively low $g^{\left(2\right)}\left(0\right)=0.045\pm 0.005$, as shown in Figure 12b, a figure comparable to results observed with EQDs operating at cryogenic temperatures [98,100]. In contrast, room-temperature single-photon sources (SPSs) employing alternative materials, such as NV centers [101] or individual organic molecules [102], encountered significant background noise issues.

## 5. Precise Positioning of Single Quantum Dots

As discussed in the previous section, precise positioning of quantum dots is crucial for practical applications, especially when integrated with cavities. Fortunately, solution-processed colloidal quantum dots offer significant flexibility for such positioning operations. The following will review efforts at precise positioning of colloidal quantum dots, including approaches for large-scale preparation.

**Figure 13:**
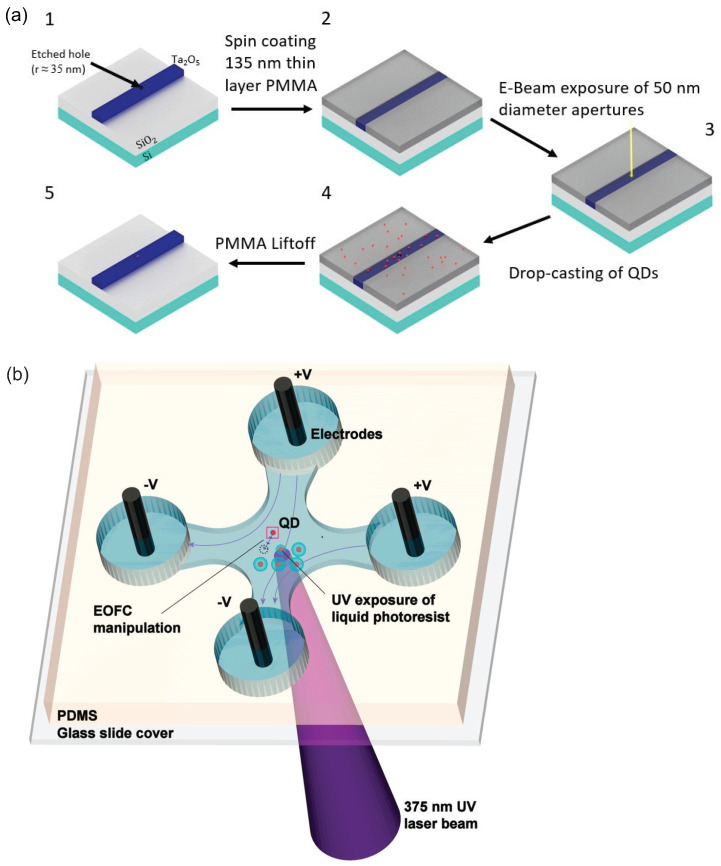
(**a**) Schematic illustration of the integration of CQDs with ${\mathrm{Ta}}_{2}O_{5}$ waveguides [103]. Reproduced with permission from Reference [103]. Copyright 2022 American Chemical Society. (**b**) Schematic diagram of EOFC microfluidic device [104]. Reproduced with permission from Reference [104]. Copyright 2010 American Chemical Society.

Figure 13a illustrates a fabrication process designed to integrate a single CQD into a nanophotonic circuit [103]. In this study, a ${\mathrm{Ta}}_{2}O_{5}$ photonic waveguide with a 35 nm radius hole was initially prepared. Subsequently, a sacrificial PMMA layer was spin-coated onto the substrate. Using electron beam lithography, a 50 nm aperture was then created at a designated position on the PMMA film. Diluted CQDs in a decane solution were subsequently drop-cast onto the sample. The final step involved removing the sacrificial PMMA layer, taking with it the excess emitters. Through multiple iterations of this procedure, the efficiency of placing a single CQD in a predefined position for coupling with nanophotonic circuits approached near-perfection.

Electroosmotic flow control (EOFC) offers another approach for achieving precise positioning of individual CQDs [104]. As depicted in Figure 13b, the CQDs were dispersed in a water-based, negative-tone photoresist. This mixture then filled a thin sheath within a cross microfluidic channel. Electrodes positioned at each end of the channel supplied the necessary voltages for EOFC, allowing for two-dimensional manipulation of CQDs at the channel surface. Through real-time imaging of the microfluidic device, instantaneous positioning of a chosen single CQD was monitored. The voltages across the four electrodes were adjusted in tandem to guide the CQD to its intended position. Once the selected CQD attained its target location, the surrounding fluid underwent brief exposure to a concentrated ultraviolet light beam, facilitating polymerization and thus immobilizing the chosen CQD. The average deviations in the final positions of the CQDs, post-immobilization, were approximately 155 nm from their target locations. By using multiphoton absorption polymerization techniques, polymerized regions can be minimized to achieve diameters under 100 nm [105,106].

**Figure 14:**
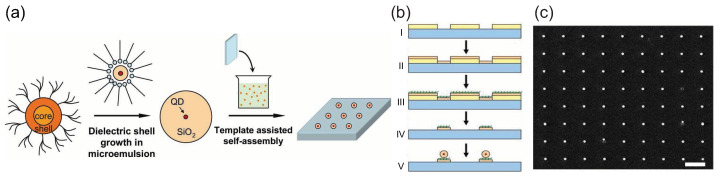
The fabrication process flow of highly ordered 2D arrays of SPSs based on CdS CQDs. (**a**) CQDs are encapsulated in the silica shell and then self-assemble into 2D arrays after template-assisted self-assembly. (**b**) The fabrication of templates with a highly ordered array of charged pads and the settlement of enlarged CQD particles on these pads by electrostatic force. (**c**) SEM image of a highly ordered array of single silica-clad CQDs formed by electrostatic force self-assembly [107]. Reproduced with permission from Reference [107]. Copyright 2008 Optica Publishing Group.

For large-scale photonic quantum technology applications, highly ordered 2D arrays of SPSs are desired. An approach based on CdS CQDs was demonstrated by Zhang et al. [107], with the fabrication process flow detailed in Figure 14. Initially, smaller CQDs were enveloped in a thick (∼100 nm) optically transparent silica shell. Subsequently, well-ordered 2D arrays of diminutive pads, tailored to match the size of the enlarged CQD particles, were fabricated on a silicon substrate using deposition and lift-off processes. The key for the self-assembly of these enlarged CQD particles lies in the static interactions between the CQDs’ silica shell and the oppositely charged polyelectrolyte pads [108]. Concluding the process, the sample showcasing the array of small pads was submerged in an ethanol solution loaded with negatively surface-charged, enlarged CQD particles, effectively capturing these charged entities. A typical single silica-clad CQD exhibited a $g^{\left(2\right)}\left(0\right)$ of 0.101 at room temperature. Even though a thicker shell is mandated in this procedure, it is noteworthy that controlling nanocrystals with diameters less than 50 nm at predetermined chip locations is achievable through various techniques [109,110].

## 6. Conclusions

This review discusses recent achievements of CQDs as SPSs, a crucial component of quantum photonics. An overview of the optical properties of CQDs is provided, and various SPS devices based on CQDs are presented. CQDs are exceptional fluorescent materials at room temperature. Through the control of their structure, they can emit highly pure and polarization-controllable single photons within desired wavelength ranges. Additionally, the solution-based processes facilitate their integration with various photonic structures through nano-fabrication techniques, enhancing single-photon collection efficiency or improving luminescent properties. These capabilities make CQDs well suited for applications in photonic quantum technologies such as quantum cryptography. It is important to note that certain quantum information technology applications necessitate the use of indistinguishable photons [7,111]. However, the spectral broadening at room temperature complicates the use of such single-photon sources in these scenarios. Additionally, even at low temperatures, CQDs encounter the issue of spectral diffusion. This phenomenon, characterized by random spectral drifts over time, hinders the production of indistinguishable photons. Exciting progress has been made recently on this issue [112], and solutions are expected with future improvements in material systems and the incorporation of microcavities. Since this review primarily concentrates on room-temperature applications, where spectral diffusion is largely overshadowed by phonon broadening, its impact on device performance remains unapparent. Therefore, an in-depth analysis of spectral diffusion is not covered in this review, but interested readers can refer to References [113,114,115] for more information. Nevertheless, it is essential to recognize that, while CQDs offer easy synthesis, greater efforts must be dedicated toward advanced integration and fabrication compared to EQDs.

## Data Availability

Not applicable.

## Abbreviations

The following abbreviations are used in this manuscript:

CLC	Cholesteric liquid crystal
cQED	Cavity quantum electrodynamics
CQD	Colloidal quantum dot
EOFC	Electroosmotic flow control
EQD	Epitaxial quantum dot
ETL	Electron-transport layer
FWHM	Full width at half maximum
HBT setup	Hanbury Brown and Twiss setup
HTL	Hole-transport layer
IRF	Instrument response function
NV	Nitrogen-vacancy
PL	Photoluminescence
PNR	Plasmonic nanoresonator
QD	Quantum dot
QY	Quantum yield
SEM	Scanning electron microscope
SPD	Single-photon detectors
SPS	Single-photon source
TEM	Transmission electron microscopy
